# Survival and *Hsp70* Gene Expression in *Plutella xylostella* and Its Larval Parasitoid *Diadegma* *insulare* Varied between Slowly Ramping and Abrupt Extreme Temperature Regimes

**DOI:** 10.1371/journal.pone.0073901

**Published:** 2013-09-06

**Authors:** Md Habibullah Bahar, Dwayne Hegedus, Juliana Soroka, Cathy Coutu, Diana Bekkaoui, Lloyd Dosdall

**Affiliations:** 1 Agriculture and Agri-Food Canada, Saskatoon Research Centre, Saskatoon, Saskatchewan, Canada; 2 Department of Agricultural, Food and Nutritional Science, University of Alberta, Edmonton, Alberta, Canada; Fred Hutchinson Cancer Research Center, United States of America

## Abstract

**Background:**

In nature, insects have evolved behavioural and physiological adaptations to cope with short term exposure to extreme temperatures. Extreme heat events may increase as a result of climate change; this in turn will affect insect population dynamics. We examined the effect of abrupt and ecologically relevant gradual exposure to high temperatures on the survival and *hsp70* gene expression in diamondback moth (DBM) adults and the parasitoid *Diadegma* *insulare* , as well as in parasitized and non-parasitized DBM larvae.

**Principal Findings:**

Tolerance to high temperatures in DBM adults was higher than in *D* *. insulare* adults. There was no difference in the survival of DBM adults between abrupt and ramped increases from 25 to 38°C; however, at 40°C survival was higher when the temperature increased gradually. In contrast, more *D* *. insulare* adults survived when the temperature was ramped rather than shifted abruptly to both 38 and 40°C. There was no heat stress effect of up to 40°C on the survival of either parasitized or non-parasitized DBM larvae. In adults of both species, more *hsp70* expression was observed when temperatures increased abruptly to 38°C compared to ramping. In contrast, at 40°C significantly more expression was found in insects exposed to the ramping rather than the abrupt regime. *Hsp70* expression level was in agreement with adult survival data and appears to be a good indicator of stress levels. In parasitized and non-parasitized larvae, *hsp70* expression was significantly higher after abrupt shifts compared to ramping at both temperatures.

**Conclusions/Significance:**

*Hsp70* gene expression was responsive to extreme temperatures in both DBM and *D* *. insulare* , which may underlie the ability of these insects to survive in extreme temperatures. Survival and *hsp70* expression upon abrupt changes are distinctly different from those after ramping indicating that experimental protocol must be considered before extrapolating laboratory results to natural field situations.

## Introduction

One of the features of global climate change is the increasing magnitude and frequency of extreme temperature events [1]. Daily thermal amplitudes (the difference between nocturnal minimum and diurnal maximum) are expected to vary greatly along latitudinal and altitudinal gradients [2]. Living organisms will need to adapt to such extreme temperature variations to avoid extinction [3]. Due to their ectothermic nature, insects are highly vulnerable to extreme and fluctuating temperatures [4]; therefore, study of the impact of short term extreme temperatures on insects is important as temperature has the potential to severely alter pest/predator/parasitoid dynamics.

Several studies have examined the phenotypic and physiological responses of insects exposed to extreme temperatures; however, most of these studies have been done by exposing the subjects to sudden changes in extreme temperatures. In nature, daily temperature fluctuations are gradual and it is crucial to study the effect of extreme temperatures in a more ecologically relevant manner [5–7]. The experimental protocol can influence the determination of thermal limits [8], for example Chown et al. [9] found variable thermal limits for *Drosophila melanogaster* exposed to different rates of thermal change. Our previous study showed significant differences in the development of diamondback moth (DBM), *Plutellaxylostella* (L) (Lepidoptera: Plutellidae), and its parasitoid *Diadegma* *insulare* (Cresson) (Hymenoptera: Ichneumonidae) between constant and fluctuating temperature regimes [10].

The molecular mechanisms underlying the adaptation to high and low extreme temperatures by insect pests and their natural enemies are garnering increasing interest [11]. The heat shock protein 70 (hsp70) family is a highly conserved group of intracellular proteins, the synthesis of which is increased upon heat shock or exposure to other environmental stresses [12]. In general, genes encoding hsp are expressed at very low levels under normal conditions, but their expression increases rapidly in response to a stress [13]. Heat shock proteins protect the cell by preventing irreversible denaturation of proteins at high temperature; therefore, *hsp70* gene expression is considered to be a potential biomarker indicating response to changing climate [14,15]. *Hsp70* gene expression has been examined in several insects, but a few experiments used slowly increasing or decreasing temperature regimes.

DBM is the most destructive insect pest of crucifer crops worldwide [16]. In North America, *D* *. insulare* is the principal natural enemy of DBM, having an efficient host-searching capability that can significantly control DBM populations [17,18]. As such, the physiological and molecular responses of DBM and its major natural enemy to potential climate change should be examined. The study by Sonoda and Tsumuki [15] is apparently the only work to quantify *hsp70* gene expression in DBM, which was conducted on an Asia-Pacific population of the moth; no published study has examined *hsp70* gene expression in the parasitoid *D* *. insulare* . During the canola growing season (May to September) in Saskatchewan, Canada, the amplitude between the maximum and minimum temperature is 14°C with extreme temperature reaching as high as 40°C [19]. Insects may be more likely to survive these extreme high temperatures if they are afforded an opportunity to adapt during a gradual increase in temperature. The aim of this study was to determine the survival of North American DBM and *D* *. insulare* exposed to different short term extreme temperature regimes (abrupt or ramped shifts to 38 or 40°C) and to quantify *hsp70* gene expression to understand the underlying mechanism for surviving in conditions of frequent extreme temperature events.

## Materials and Methods

### Insects

DBM and *D* *. insulare* were maintained in a laboratory growth cabinet with six to eight week-old canola plants at 25 ± 1 °C and 70% RH, and a photoperiod of 16:8 (L: D) hours. DBM larvae fed on canola plants and were parasitized by *D* *. insulare* . Adults of both insect species were provided 10% honey-sugar solution which was administered via a dental roll in 35 ml plastic cups (Solo Cup Company). DBM larvae were collected periodically from the research farm of Agriculture and Agri-Food Canada, Saskatoon Research Centre and added to the colonies in order to maintain genetic vigor.

### Thermo-tolerance of DBM and *D. insulare*

**Figure 1:**
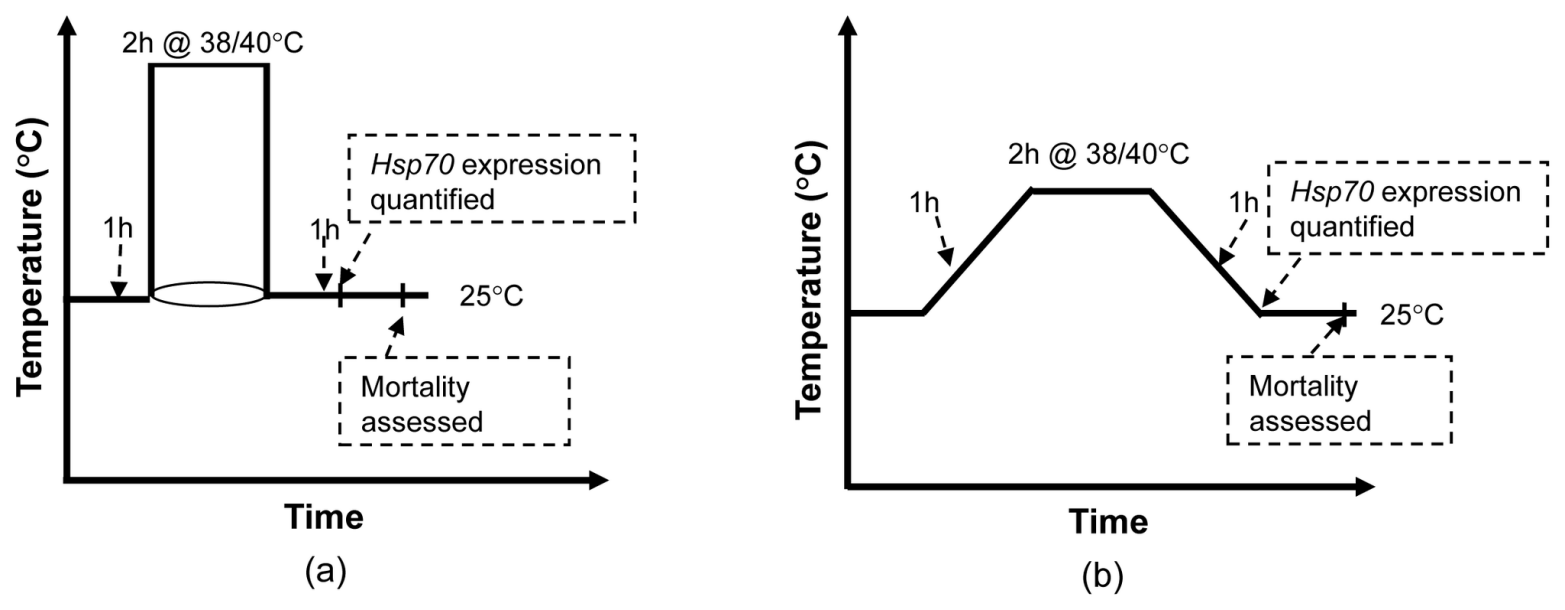
Schematic diagram of the experimental protocols. (a) insects were exposed to abrupt changes in extreme temperature (38 or 40°C) for two hours, (b) temperature was gradually increased from the control temperature to the extreme temperature (38 or 40°C), remained there for two hours and was then decreased to the control temperature (25°C).

Ten newly emerged DBM or *D* *. insulare* adults were placed in individual plastic cup (6 × 4 cm^2^) along with a dental roll moistened with distilled water and transferred to an incubator where they were acclimated at 25°C for 24 hours. Following acclimation, the insects in their cups were transferred to individually controlled thermal gradient cells [10] where they were exposed to different temperature regimes: an abrupt shift to 38 or 40°C or ramping (slowly increasing at a steady rate over one hour) to 38 or 40°C, at which they were held for two hours. A schematic diagram of the protocol is provided in the Figure 1. These two extreme temperatures (38 and 40°C) were selected based on a preliminary experiment on the impact of temperature on the survival and development of DBM and *D* *. insulare* (data not shown) as well as on the basis of historical weather data of the province Saskatchewan, Canada. After the heat treatment, the insects were immediately transferred back to the 25°C incubator (rearing temperature) for two hours at which time survival was evaluated. Insects that failed to move an appendage after probing with forceps were considered dead. As a control, ten adults were handled in a similar manner as the test insects, but were kept in the incubator at 25°C for the duration of the experiment. Fifteen replications of each treatment and control were conducted.

A similar protocol was followed for 4^th^ instar parasitized and non-parasitized DBM larvae with the exception that larvae were placed in plastic Petri dishes (5.5 cm) fitted with moistened filter paper. To obtain fourth instar parasitized DBM larvae, second instar DBM larvae were placed on canola leaf tissue in plastic Petri dishes (10 cm) fitted with moistened filter paper. One mated female *D* *. insulare* adult was released in each Petri dish and observed until parasitization occurred. Following parasitization, DBM larvae were reared on canola leaves in plastic Petri dishes (5.5 cm) at 25°C until they reached the fourth instar.

### Quantification of heat shock protein gene (*hsp70*) expression

Ten DBM adults or 15 *D* *. insulare* adults were placed in individual 13 ml plastic vials (16.5 × 10.0 mm, Starstedt Inc., Newton, NC, USA) and exposed to the temperatures described for survival studies or the control temperature of 25°C. After heat treatment and one hour of recovery time at 25°C, insects were frozen in liquid nitrogen and stored at -80°C. Each treatment was replicated three times for DBM and four times for *D* *. insulare* . A similar protocol was followed for parasitized and non-parasitized fourth instar DBM larvae, but with four larvae in 2 ml micro-centrifuge tubes (Starstedt Inc., Newton, NC, USA). Each treatment was replicated three times.

### Designing primers for RT–qPCR

To design primers for RT–qPCR (Table 1), one µg mRNA from both DBM and *D* *. insulare* adults as well as parasitized and un-parasitized DBM larvae was sequenced on a Roche 454 GS FLX Titanium at the DNA Technology Unit of National Research Council of Canada, Saskatoon. *De novo* assembly of reverse transcribed mRNA sequence reads was performed using CLC genomics workbench 5.5.1 (*CLC bio*, Cambridge, MA, USA). BLASTN was performed with a DBM heat shock inducible protein gene sequence (*hsp70*) (accession AB325801.1) on the assembled transcripts in the database to identify the DBM allele in our colony. The predominant allele in our colony has several single nucleotide polymorphisms that prevented amplification using primers designed previously for AB325801.1 [15]. After BLASTN, RT–qPCR primers for DBM *hsp70* were designed for the top hits (>90% identical to AB325801.1 at nucleotide level). Similarly, RT–qPCR primers for *D* *. insulare* *hsp70* were designed for the top hits (>75% identical to AB325801.1 at nucleotide level) from *de novo* assembly of RNA sequence reads for *D* *. insulare* adults. The DBM and *D* *. insulare* *hsp70* genes identified in this study have been deposited in GenBank as KC841275.1 and KC841277.1 respectively.

**Table 1:** Primers used for target genes and reference genes in RT–qPCR.

**Gene**	**Direction**	**Primer sequences (5'–3')**	**Amplicon size (bp)**	**GC%^1^**	**Tm^2^ (°C)**
*D* *. insulare* * hsp70*	Forward	CGAAACGGGCCCTCGAAACC	122	65	66
	Reverse	GATTGAGAACAGCGGCTGAG		55	62
*D* *. insulare* β-actin	Forward	TCCGATCGAGCACGGAATCG	136	60	64
	Reverse	TCGCTTTTGGGTTCAATGGC		50	60
DBM *hsp70*	Forward	CGTTGAGGAGGCTACGAACC	145	60	64
	Reverse	CGCGTTCAGCTCTTCGAACC		60	64
DBM β-actin	Forward	TGTTCGAGACCTTCAACACG	128	50	60
	Reverse	AGATGGGCACGGTGTGGGAG		65	66

^“1″^: GC%, Proportion of Guanine and Cytosine.

^“2″^: Tm. Annealing temperature.

To design β–actin primers (housekeeping reference gene used for normalizing the data) for DBM, BLASTN was performed with the DBM actin (accession JN410820) against the assembled DBM adult and larval sequences and primers were designed from the top hit. For β–actin primers of *D* *. insulare* , BLASTN was done with *Spodoptera* *frugiperda* actin (accession HQ008727) against the assembled *D* *. insulare* adult sequences and primers were designed from the top two hits. The DBM and *D* *. insulare* β–actin genes identified in this study have been deposited in GenBank as KC841274.1 and KC841276.1 respectively.

Primer pairs were predicted to produce unique amplicons of no more than 150 bp in length, with a GC content of 50-65% and an annealing temperature (Tm) of 60-66°C. Product size was confirmed by PCR followed by 1% agarose gel electrophoresis. Primers were validated by melt curve analysis and PCR efficiency was evaluated by establishing a standard curve with eight 10-fold dilutions [20]. Since parasitized DBM larvae also contain *D* *. insulare* tissue, a PCR analysis was performed using both sets of primers and cDNA from both insects to confirm that they could discriminate between DBM and *D* *. insulare* *hsp70* and β-actin genes.

### Real-time quantitative PCR (RT–qPCR)

Total RNA from whole insects was extracted using Illustra RNAspin Mini Kit (GE Healthcare) according to the manufacturer instructions. The quality and quantity of RNA were determined by 1.2% agarose gel electrophoresis and using a NanoDrop Spectrophotometer, respectively (Thermo Fisher Scientific Inc, Waltham, MA, USA). One µg of RNA was reverse transcribed into single strand cDNA using 5 × iScript^TM^ Reverse Transcription Supermix for RT–qPCR (Bio-Rad Laboratories, Inc, Hercules, CA, USA).

The RT–qPCR was conducted on a thermal cycler (Bio-Rad C1000^TM^ Thermal Cycler, CFX96^TM^ Real-Time System). Each PCR reaction was run in triplicate in a 20 µL total reaction volume comprising 6µL cDNA (300 ng), 1µL (5 µM) of each primer, 2 µL of DEPC water and 10 µL of SsoFast^TM^ EvaGreen® Supermix (Bio-Rad Laboratories, Inc, Hercules, CA, USA). The qPCR cycling parameters were 98°C for 2 min, followed by 40 cycles of 98°C for 5 s, 55°C for 5 s and then 95°C for 10 s, and finally a melt curve from 65 to 95°C with an increment of 0.5°C per 5 s. The relative level of *hsp70* transcript was defined as the fold amount compared to the amount of β-actin using Bio-Rad CFX Manager^TM^ (Bio-Rad Laboratories, Inc, Hercules, CA, USA). A control level of *hsp70* at 25°C was quantified in each plate to correct for variation within the plate. The transcript levels in each treatment were expressed relative to the constant 25°C which was set at 1. Each reaction was performed three times with three to four biological replications.

### Statistical analysis

A GLZ (Generalized Linear Model) analysis was done on survival data using SAS 9.3 PROC LOGISTIC [21]. One-way ANOVA was performed to analyze *hsp70* gene expression among treatments using the statistical software “R” [22]. Multiple comparisons among the treatments were done using Tukey’s HSD test.

## Results

### Survival

**Figure 2:**
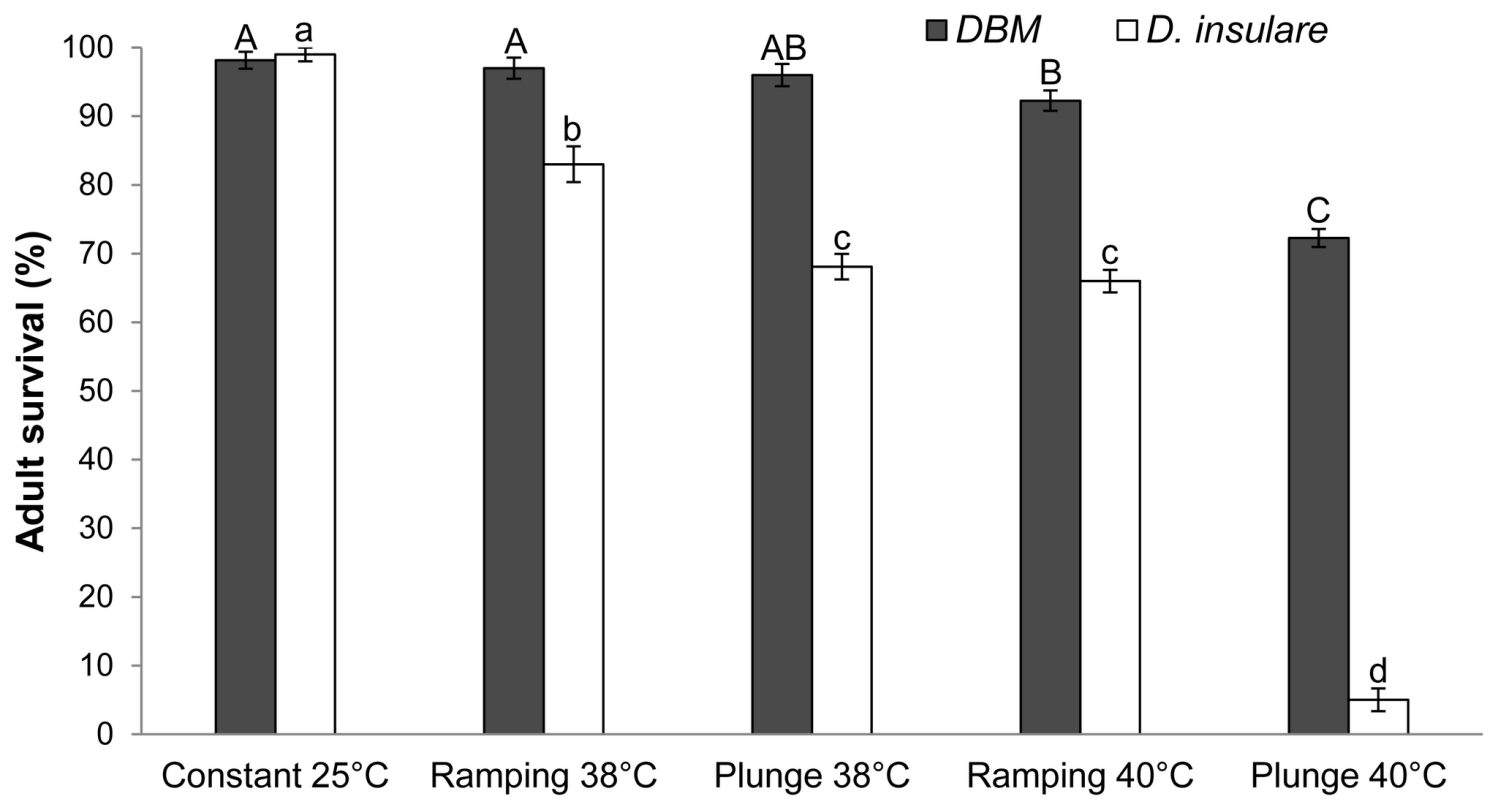
Survival (%±SE) of diamondback moth (DBM) and *Diadegma insulare* at constant control (25°C) or after an abrupt or slow shift to an extreme temperature followed by two hours of recovery time. Same letters above the solid (DBM) or open (* D. insulare *) bars indicate no significant differences (α = 0.05).

There were significant effects of both temperature *per se* (*P* < 0.001) and temperature protocol (*P* < 0.001) on the survival of *D* *. insulare* adults. Significantly more *D* *. insulare* adults survived when the temperature was ramped compared to an abrupt shift at both temperatures (at 38°C, *P* < 0.001; at 40°C, *P* < 0.001) (Figure 2). In the case of DBM adults, temperature also significantly (*P*<0.001) affected the survival. At 40°C, significantly (*P*<0.001) more DBM adults survived after the ramping treatment than the abrupt shift treatment, while there was no such difference between the protocols at 38°C (*P* = 0.52) (Figure 2). The survival of *D* *. insulare* adults was significantly reduced at both temperatures and more so than that of DBM. There was no effect of temperature or temperature protocol on the survival of either parasitized or non-parasitized larvae as all larvae survived at all temperature regimes.

### Heat shock protein (*hsp70*) gene expression in adults

**Figure 3:**
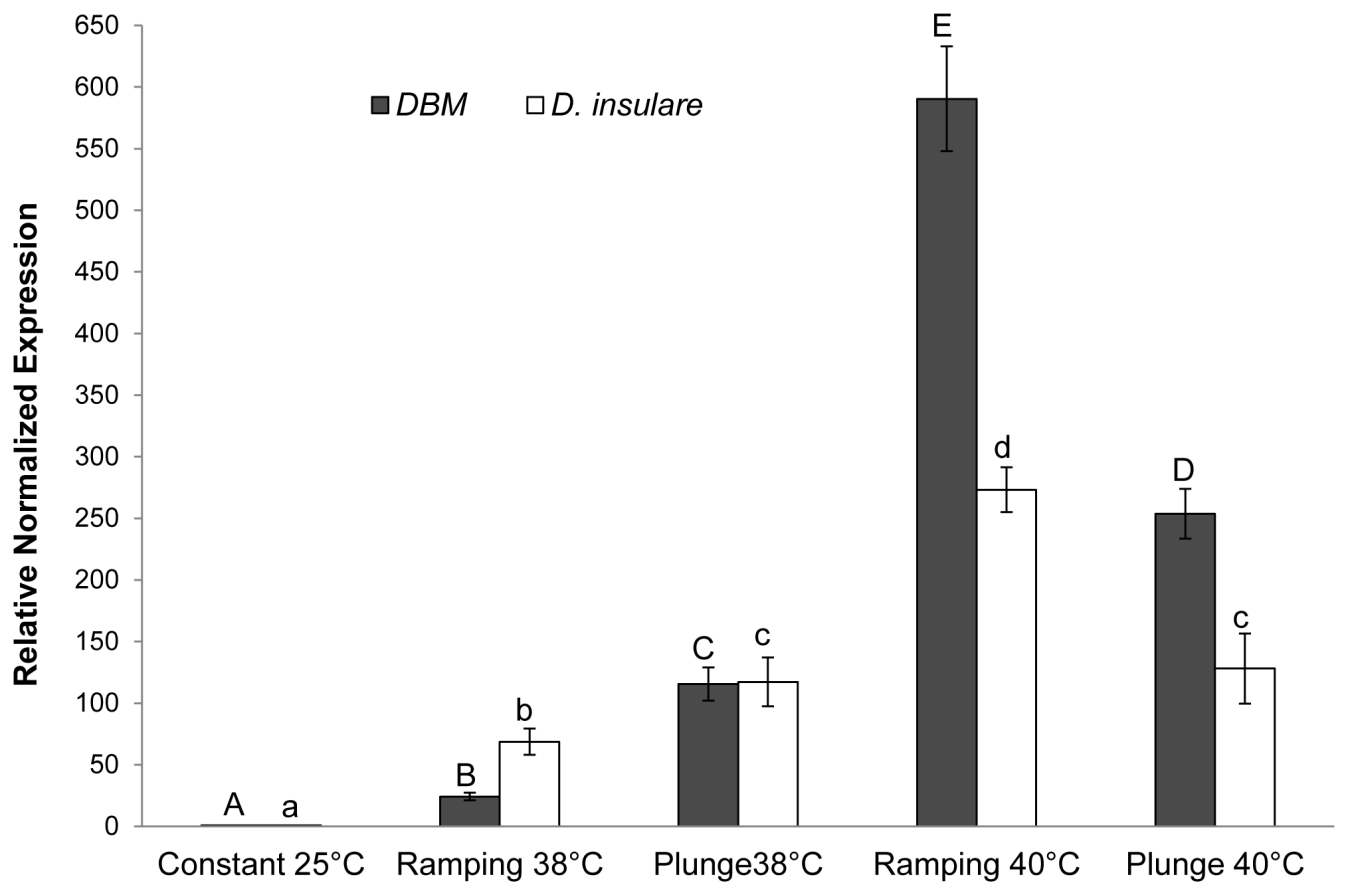
*Hsp70* expression in diamondback moth (DBM) and *Diadegma* *insulare* adults under various tempertaure regimes. Relative normalized expression indicates the level of transcripts normalized to the internal standard (β-actin). The levels in treatments are expressed relative to the constant 25°C set at 1. Each bar represents the mean ± SE of three independent experiments for DBM and four independent experiments for *D* *. insulare* . Same letter above in solid (DBM) or open (* D. insulare *) bars indicate no significant difference (α = 0.05).

*Hsp70* gene expression in adults of both DBM and *D* *. insulare* was significantly (DBM, ANOVA, *P* < 0.001, *F* = 121.2, *df* = 4, 10; *D* *. insulare* , ANOVA, *P* < 0.001, *F* = 30.6, *df* = 4, 15) up-regulated at all extreme temperature regimes compared to the constant 25°C control. In both insects, there was significantly more relative *hsp70* expression when the temperature was shifted abruptly to 38°C than ramping to 38°C (DBM, Tukey’s HSD, *P* = 0.003; *D* *. insulare* , Tukey’s HSD, *P* = 0.07). In contrast, at 40°C more relative expression of the *hsp70* gene was found with the ramping temperature regime than the abrupt shift (DBM, Tukey’s HSD, *P* = 0.002; *D* *. insulare* , Tukey’s HSD, *P* = 0.005) (Figure 3). In DBM adults, there was a significant difference in *hsp70* gene expression between ramping to 38°C and ramping to 40°C (Tukey’s HSD, *P* < 0.001), as well as between abrupt shift to 38°C and 40°C (Tukey’s HSD, *P* = 0.008). Conversely, in *D* *. insulare* adults, although there was significantly higher expression with ramping to 40°C than ramping to 38°C (Tukey’s HSD, *P* < 0.001), there was no difference between the abrupt shift to 38°C or 40°C (Tukey’s HSD, *P* = 0.99) (Figure 3).

### Heat shock protein (*hsp70*) gene expression in larvae

**Figure 4:**
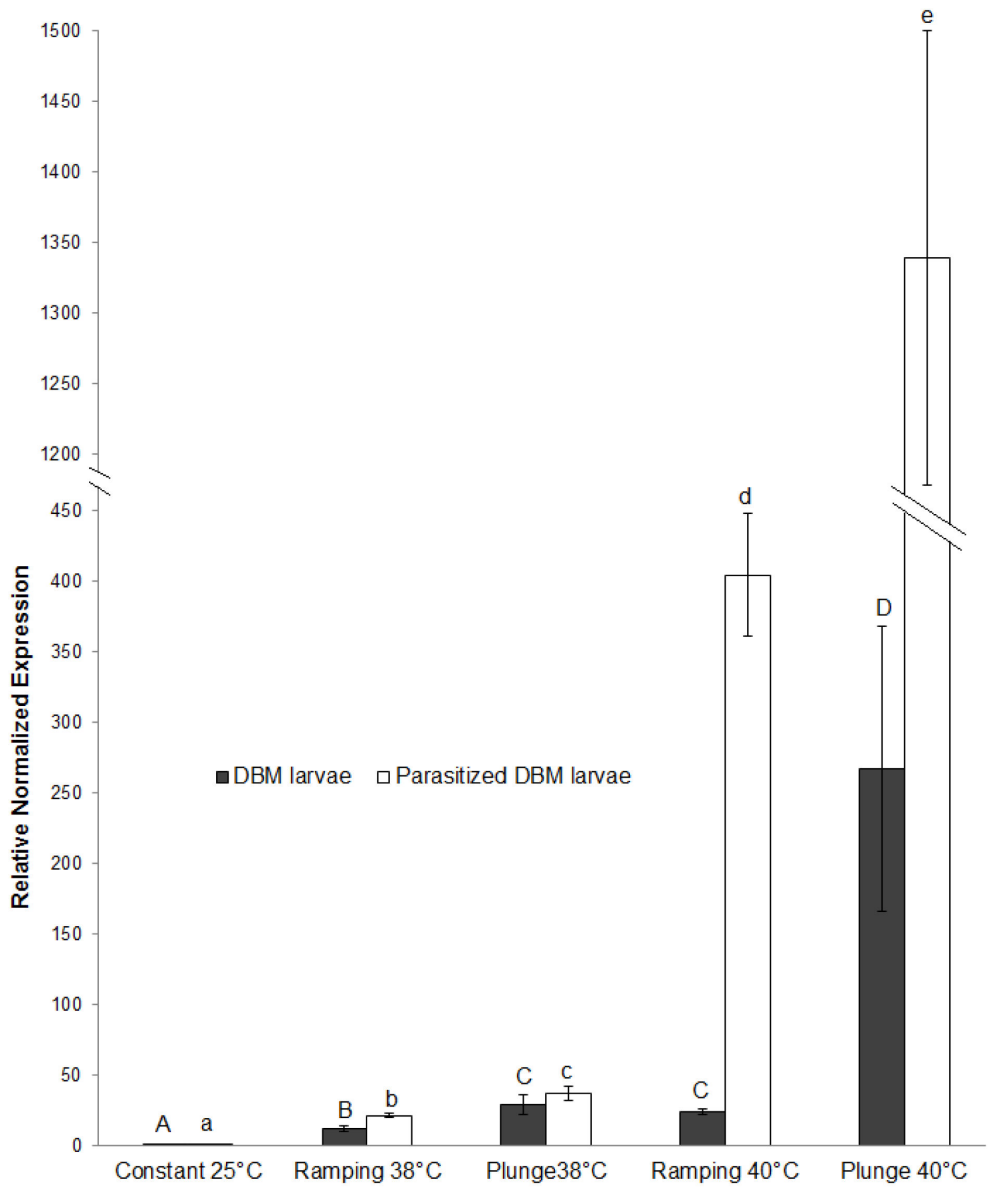
*Hsp70* expression in parasitized and non-parasitized diamondback moth (DBM) larvae and parasitized DBM larvae under various tempertaure regimes. Relative normalized expression indicates the level of transcripts normalized to the internal standard (β-actin). The levels in treatments are expressed relative to the constant 25°C set at 1. Each bar represents the mean±SE of three independent experiments. Same letter above solid (non-parasitized DBM larvae) or open (parasitized DBM larvae) bars indicate no significant difference (α = 0.05).

Like adults, *hsp70* gene expression in both parasitized and non-parasitized DBM larvae was significantly (non-parasitized larvae, ANOVA, *P* < 0.001, *F* = 59.4, *df* = 4, 10; parasitized larvae, ANOVA, *P* < 0.001, *F* = 58.8, *df* = 4, 10) up-regulated in all extreme temperature regimes compared to the constant 25°C control. In both larvae at both temperatures, there was significantly more relative expression of the *hsp70* gene with the abrupt shift regime than ramping (non-parasitized DBM larvae, at 38°C, Tukey’s HSD, *P* = 0.008 and at 40°C, Tukey’s HSD, *P* < 0.001; parasitized DBM larvae, at 38°C, Tukey’s HSD, *P* = 0.02 and at 40°C, TukeyHSD, *P* = 0.03). In parasitized DBM larvae, there was a significantly higher *hsp70* gene expression at ramping to 40°C than ramping to 38°C (Tukey’s HSD, *P* < 0.001), and at abrupt shift to 40°C than to 38°C (Tukey’s HSD, *P* < 0.001). On the other hand, in non-parasitized DBM larvae there was significantly higher expression after an abrupt shift to 40°C than to 38°C (Tukey’s HSD, *P* < 0.001), but there was no difference between ramping to 38°C and ramping to 40°C (Tukey’s HSD, *P* = 0.99) (Figure 4).

## Discussion

The major findings in this study were the variation in survival and *hsp70* gene expression in DBM and *D* *. insulare* between abrupt and slowly ramping to extreme high temperatures. Survival of adult DBM at 40°C and *D* *. insulare* at 38 and 40°C was greater when the temperature increase was gradual instead of abruptly changed. Higher survival after gradually increasing to extreme temperature suggests that slower rates of temperature change allow for some form of induced adaption that allows the insect to better survive the elevated temperatures. The abrupt shift would not permit sufficient time for this to occur and the insect will be exposed to the extreme temperature in a naïve, unprepared physiological state. Our results agree with several previous works which showed that slower rates of temperature increase enhance subsequent heat tolerance of insects [5,6,9,23,24]. This further emphasizes that experimental protocol can significantly influence the phenotypic and physiological responses of insects. Apart from the control treatment, DBM survived better than its parasitoid *D* *. insulare* at the two extreme temperatures. Lower survival of *D* *. insulare* adults than DBM adults at extreme temperatures indicates that *D* *. insulare* is less thermo-tolerant than DBM, which agrees with our previous study [10]. It is generally established that parasitoids possess a narrower thermal tolerance than their hosts [25,26] which has profound implications for host-parasitoid population dynamics. It is conceivable that more frequent and more severe extreme temperature events that are predicted to occur due to climate change may sway the dynamic in favour of DBM. This may in turn lead to longer and more involved pest outbreaks should natural control mechanisms be compromised.

Insect thermo-tolerance is correlated with *hsp70* gene expression [27,28]. Our experiments focused on the quantification of *hsp70* gene expression during short term exposure to extreme temperatures attained slowly through ramping or abruptly. In adults of both species, *hsp70* gene expression was higher after an abrupt temperature shift to 38°C than after ramping, indicating that the insects are under greater stress during rapid temperature shifts. At 40°C, survival of both adults was significantly higher after ramping than after an abrupt shift and the increased survivorship with the ramping protocol was correlated with *hsp70* gene expression at this temperature. Slowly increasing the temperature might allow for acclimation to the high extreme temperature and hence, increased heat shock tolerance. In *Drosophila melanogaster*, natural increase of temperature is positively correlated with increased heat tolerance [29] and defence against heat stress is consistently associated with the up-regulation of *hsp* gene expression [30]. At the higher extreme temperature, slowly increasing temperature may also allow individuals to continuously express *hsp70* at high levels. For example, in *Tenebrio* *molitor* L. (Coleoptera: Tenebrionidae) *hsp70* gene expression was higher at fluctuating temperatures (-2.5 to 43°C) than under constant temperature (18°C) with the same average temperature [31]. It is also possible that once the adult insect’s extreme threshold temperature is reached, as indicated by the increased mortality, the effect on insect physiology is different than at the lower extreme temperature. In DBM adults, *hsp70* gene expression was higher after an abrupt shift to 40°C than to 38°C, whereas in *D* *. insulare* there was no difference. The overall heat tolerance of DBM is higher than *D* *. insulare* [10] and the latter likely had already reached its upper threshold level at 38°C leading to mortality and no differences in *hsp70* gene expression. These findings again support the statement that *hsp70* gene expression can be correlated with tolerance to stress temperatures [13].

Similar the situation with adults, in either parasitized or non-parasitized DBM larvae there was a significant difference in *hsp70* expression between the abrupt shift and ramping protocols. In larvae at both 38°C and 40°C, more *hsp70* gene expression was observed after the abrupt shift treatment than the ramped treatment. At 40°C, the pattern for larvae was the opposite of that for adults where higher *hsp70* gene expression was observed after ramping than after an abrupt shift in temperature. This discrimination is not unexpected as temperature thresholds may vary across life stages and sexes. Indeed, larvae are more thermo-tolerant than adults in most arthropods [32,33]. For instance, *hsp70* gene expression was higher in adults than larvae of *Macrocentruscingulum* (Hymenoptera: Braconidae) [34]. In our study, survival of adult DBM was significantly reduced after abrupt change to 40°C, while all the larvae survived this extreme temperature. Gender may also affect thermo-tolerance as *hsp70* gene expression was higher in males than in females of the butterfly, *Lycaena* *tityrus* (Poda) [35].

Hsp70 proteins act as molecular chaperones to protect an organism from heat and related stresses and have been proposed as potential biomarkers of stress tolerance [14,15]. Global climate change is gradual rather than abrupt; however, it is predicted to be accompanied by frequent and more extreme temperature fluctuations [1]. In this study, *hsp70* gene expression was responsive to heat treatment in DBM and its larval parasitoid *D* *. insulare* . The sensitivity of *hsp70* gene expression to extreme temperature suggests this may be used to estimate an insect’s ability to adapt and survive short term exposure to extreme temperature events. However, survival of DBM was less affected by extreme temperature than *D* *. insulare* suggesting that host-parasitoid population dynamics could shift in favour of DBM should climate change lead to more frequent occurrences of elevated temperatures. Along with previous studies [8,10,31,36,37], our findings further emphasize the need to consider temperature exposure regimes when extrapolating laboratory studies to natural field situations. Such models will be far more informative and predictive if ecologically-relevant local conditions involving gradual ramping to high temperatures are considered. This study examined only two extreme temperatures based on our previous experiments on the survival and development of DBM and *D* *. insulare* at different temperatures, but we know neither at what temperature both insects fail to induce *hsp70* nor if *hsp70* gene expression varies among their life stages. Such studies will better define the thermo-tolerance in these insects throughout their life cycles and allow for better prediction of host-parasitoid dynamics and outcomes when combined with climate change models.
